# Deep vein thromboembolism after arthroscopy of the shoulder: two case reports and a review of the literature

**DOI:** 10.1186/1471-2474-11-65

**Published:** 2010-04-08

**Authors:** Raffaele Garofalo, Angela Notarnicola, Lorenzo Moretti, Biagio Moretti, Stefania Marini, Alessandro Castagna

**Affiliations:** 1 Orthopaedic and Traumatology Unit, F. Miulli Hospital, Acquaviva delle Fonti, Bari, Italy; 2 Department of Clinical Methodology and Surgical Techniques, University of Bari, Bari, Italy; 3 President of Course of Motor and Sports Sciences, Faculty of Medicine and Surgery of University of Bari, Bari, Italy; 4 Radiology Unit, F. Miulli Hospital, Acquaviva delle Fonti, Bari, Italy; 5 Shoulder and elbow unit IRCCS Humanitas Institute Milano, Milano, Italy

## Abstract

**Background:**

Deep vein thrombosis (DVT) has an incidence of 1 case per 1000 inhabitants in the general population and it is very rare after arthroscopy of the shoulder. Therefore, the current guidelines do not advise the administration of DVT prophylaxis in shoulder arthroscopy procedures.

**Cases presentation:**

We describe two cases of thrombosis of the arm after shoulder arthroscopy on a total of 10.452 shoulder arthroscopies performed during a period of ten years. One of two patients was further complicated by a bilateral pulmonary microembolism. In these two clinical cases the complication developed despite the absence of risk factors such as a concomitant neoplasm, thrombophilia, smoking habit, or a long duration of the procedure.

**Conclusions:**

The DVT after shoulder arthroscopy procedure remain a very rare complication. However, in view of the growing number of patients undergoing this procedure, this figure is expected to rise. The clinician surgeon should take in mind this possible complication that normally appears in the first 3 weeks after surgery, so to perform anti-coagulant treatment. Further clinical studies are therefore warranted to assess the true risk of VTE. In fact, the presence of "minor" predisposing factors that are not routinely studied, as well as the postoperative immobilization period, are potential risk factors that, associated with the invasiveness of the arthroscopy procedure, could trigger a thromboembolism.

## Background

Deep vein thrombosis (DVT) has an incidence of 1 case per 1000 inhabitants in the general population [1]. Only 4% of cases affect the arm because there is a lower incidence of stasis and slowing of the blood flow due to the venous circulation being located at the same level as the heart[2]. Complications associated with venous thromboebolism (VTE) of the arm include pulmonary embolism (11-26%), the superior vena cava syndrome (21-23%) and the post-thrombotic syndrome (27-50%) [3-6].

In 50% of the cases of VTE of the arm the risk factors identified are the presence of a central venous catheter or of venous compression in the thoracic outlet syndrome [7-10,3]. The remaining cases are attributable to cancer, the use of oral contraceptives, pregnancy, congenital thrombophilia, acquired coagulation defects, diabetes mellitus, obesity, smoking habit or intense sports activity [10,8,13]. Blon et al. underline the importance of assessing the risk factors considered up to now only for the legs, namely surgery, trauma, immobilization. They also demonstrate that travel, obesity, hormone therapy or childbirth do not significantly increase the risk of VTE of the upper limb [14]. In any case, the frequency of VTE often depends on the simultaneous presence of more than one risk factor and on their interaction [15].

Willis et al. [16] reported that the prevalence of DVT after reconstructive shoulder arthroplasty was 13.0%, a rate comparable to that after hip arthroplasty (10.3%) but lower than that after knee arthroplasty (27.2%). In the leg, the longer duration of the procedure and the use of lateral decubitus, blockade of the nerve plexus, as well as **i**ntravenous administration of anesthetics, traction of the limb and the use of mechanical constriction devices like thigh pumps, have all been indicated as risk factors for the onset of a VTE [16].

The current guidelines do not advise the administration of VTE prophylaxis in shoulder arthroscopy procedures, because of its minimal tissue invasiveness [1,17].

We describe two cases of thrombosis of the arm after shoulder arthroscopy, observed on a total of 10.452 shoulder arthroscopy procedures performed during the period between the 1999 and 2009. One of two patients was further complicated by a bilateral pulmonary microembolism. In our two patients, unlike the six previously reported in literature after shoulder arthrosocopy [18-21], no preoperative risk factor was present that could have predicted the onset of the thrombosis.

## Case Presentation

### Case 1

**Figure 1:**
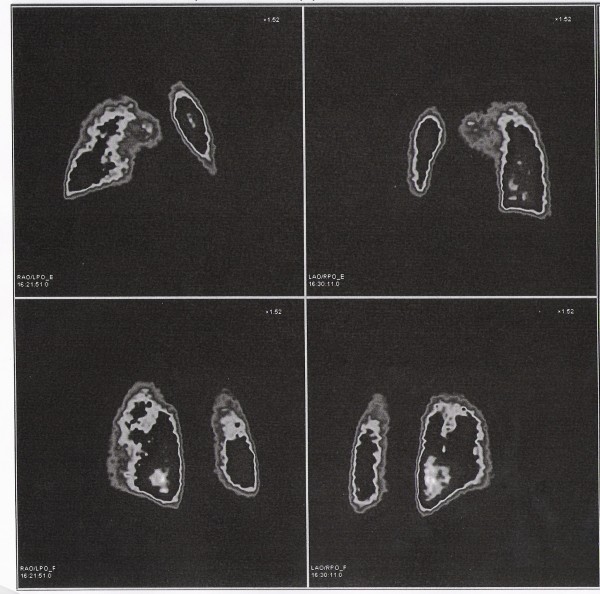
**Lung perfusion scintigraphy shows the presence of multiple perfusion defects of a sub-segmental nature and bilateral distribution, that are particularly evident in the right superior lobe, suggesting an episode of bilateral pulmonary micropolyembolism**.

A 21-year-old student (weight 75 kg, height 180 cm), non smoker and with a negative personal and family history for thrombophilia and VTE, underwent right arthroscopic capsuloplasty for recurrent glenohumeral instability. The literature suggests that the arthroscopic stabilization is a critical surgical procedure [22]. Before the procedure the patient had a Rowe score of 50/100, an SST (Simple Shoulder Test) of 10/12 and a pain (measured by a Visual Analog Scale, VAS) of 8/10. His medical history included 2 episodes of dislocation and 4 of subluxation. The arthroscopy procedure, lasting approximately 45 minutes, was done in general anesthesia, lateral decubitus and with 4 kg traction. Postoperatively, the joint was immobilized in a sling for 4 weeks, during which only active mobilization of the elbow and wrist, several times a day, were allowed. The patient did not present any neurovascular complications until 3 weeks after the procedure, when pain and diffuse swelling developed and grew progressively worse, associated with a state of anxiety and dyspnea. Blood tests showed increased values of the D-dimers (3132 ng/ml), fibrinogen (682 mg/dl), ESR (39 mm/h) and PCR (55 mg/l). Arteriovenous color-doppler of the upper and lower limbs demonstrated VTE of the humeral veins of the right arm and homolateral basilica. Lung perfusion scintigraphy revealed the presence of multiple perfusion defects of a sub-segmental nature and bilateral distribution, that were particularly evident in the superior right lobe, suggesting a bilateral lung micropolyembolism (figure 1). A VTE was diagnosed, complicated by pulmonary embolism (PE), and oxygen therapy and low molecular weight heparin (Clexane 6000 × 2/die) were administered for 6 weeks. Completion of the diagnostic work-up revealed a heterozygous mutation for the gene coding for methylenetetrahydrofolate reductase (MTHFR-C677T), but due to the absence of simultaneous defects of the factor V Leiden or factor II genes, a diagnosis of thrombophilia was excluded. After 3 weeks, blood tests and echocolor-doppler demonstrated regression of the disease. Anti-thromboembolic treatment was continued for another week and no further complication was observed at 6 months' follow-up. At the last follow-up, at one year, the patient was satisfied with the treatment, the VAS was estimated at 2/10, the Rowe score was 90/100 and the SST 3/12.

### Case 2

**Figure 2:**
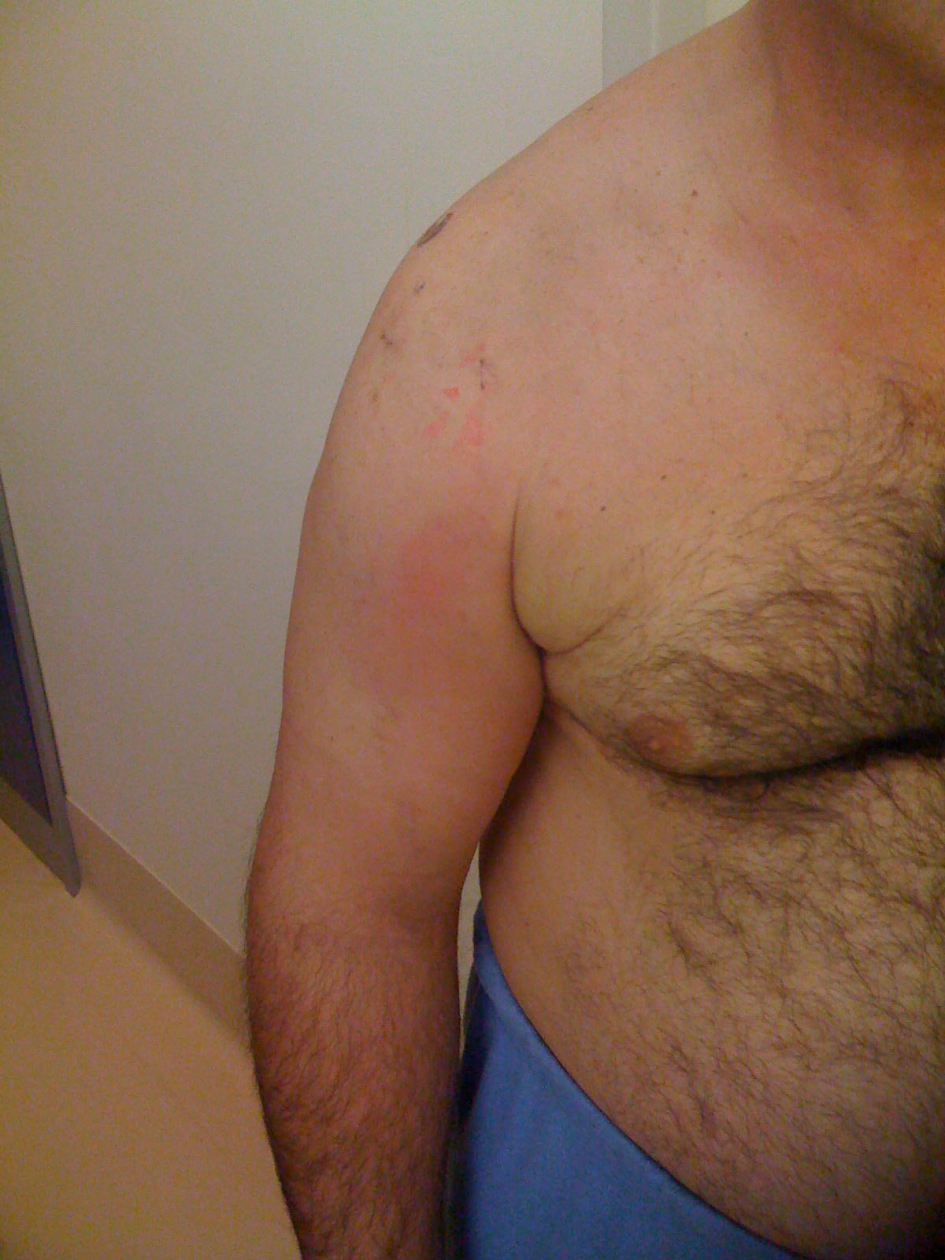
**Clinical examination 4 weeks after the shoulder arthroscopy procedure shows severe swelling of the upper right arm and axillary region**.

**Figure 3:**
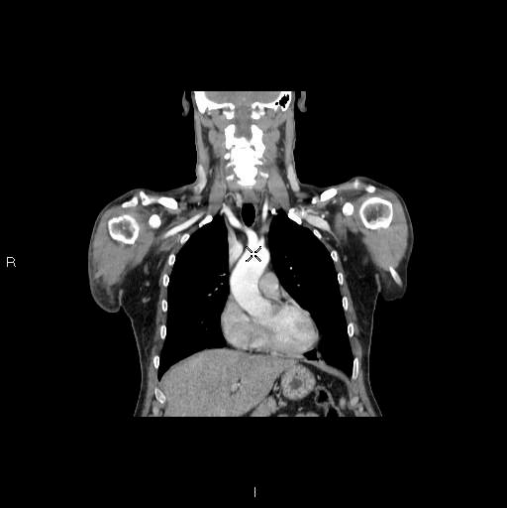
**Chest angio-CT shows diffuse thrombosis of the anonyma and subclavian veins and distal tract of the right axillary vein, without involvement of the pulmonary circulation**.

A 54-year-old male (weight 90 kg, height 178 cm), factory worker, no smoker, with a negative family history for DVT, underwent arthroscopic repair of a right rotator cuff tear. Before the procedure the patient had a Constant Score of 30/85, an SST of 9/12, a visual analog score (VAS) of 7/10. The rotator cuff repair procedure employed anchorage and sutures, and was performed in local anesthesia (plexic). The procedure lasted 50 minutes and was performed in lateral decubitus with 4 kg traction. The patient was discharged with instructions to keep the arm immobilized in the sling for 4 weeks, during which he should practise active mobilization of the wrist, elbow and shoulder several times a day. Three weeks after the procedure, the patient came to our observation with evident swelling and reddening of the axillary region and right arm, causing sharp pain (figure 2). Blood tests (D-dimers: 700 ng/ml) and arteriovenous echocolor-doppler of the arms showed a thrombus of the right anonyma vein, confirmed by chest X-ray, and excluded pulmonary involvement (figure 3). Treatment with low molecular weight heparin was administered for 10 days and then acenocumarol 4 mg/day for 2 months. After one month the oedema of the operated limb had reduced, and at 4 months the patient started to recover strength in the arm. After 8 months' follow-up he was able to return to normal working activities with no limitations. The Constant Score was then 80/85, VAS 2/10 and the SST 3/12.

## Conclusions

The risk factors for thrombosis are the same for an upper as for a lower limb, with the exception of hormone therapy, obesity and travel, that have been shown to have no significant impact [14]. Instead, a specific risk factor for the upper limbs is the presence of a central venous catheter [14]. In any case, the absolute risk of DVT is very low, and the frequency depends on the coexistence of more than one risk factor and on their interaction [15]. In fact, during the period of immobilization of the shoulder after severe trauma like glenohumeral or clavicular dislocation, or a compound fracture of the humerus [23], as also after open surgery for arthroplasty, anti-VTE prophylaxis is advised [24]. Instead, for arthroscopic procedures, the international guidelines do not advise any prophylactic treatment [1]. Few cases of VTE have been reported in literature after shoulder arthroscopy, and the authors in each case suggested the presence of concomitant acquired or congenital risk factors justifying its onset (table 1).

**Table 1:** Cases of DVT (Deep Vein Thromboembolism) described in literature after shoulder arthroscopy

*Year*	*Number of patients*	*Reference*	*Personal and family history*	*Disorder requiring shoulder arthroscopy*	*DVT event*	*Individuation of subsequent risk factors for DVT*
1988	1 case report	Burkhart SS. 1990. [20]	32-year-old male in good health	Anterior subdislocation of the left shoulder with Hill-Sachs lesion	Complete thrombosis of the left basilica and innominata veins on the 3^rd ^post-operative day	Hodgkin's lymphoma

2001	1 case report	Polzhofer et al, 2003 [19]	48-year-old male with type 2 diabetes, obesity, humeral en chondroma	Right subacromial conflict syndrome in calcific supraspinal tendinitis	Thrombosis of the right cephalic vein with pulmonary embolism on the 7^th ^postoperative day	---------

2006	1 case report	Hariri et al, 2009 [18]	25-year-old male, smoker	Glenohumeral instability	Thrombophlebitis of the brachial vein complicated by pulmonary embolism on the 10^th ^postoperative day	Prolonged duration of procedure (150 minutes), lateral decubitus and arm traction with 3 Kg, general anesthesia

2005/2006	6 DVT events on 9385 arthroscopies	Randelli et al, 2009 [17]	Italian multi-centric study	4 patients were treated for lesion of rotator cuff; 1 patient was treated for glenohumeral instability; - 1 patient was treated for acromionplasty	5 patients showed a deep vein thrombosis; 1 patient showed a pulmonary embolism	---------

2008	3 case reports	Bongiovanni et al, 2009 [21]	1° case: 30-year-old male in good health 2° case: 54-year-old female in good health 3° case: 66-year-old male in good health	1° case: Lesion of the glenoid rim and supraspinal insertion 2° case: Lesion of the rotator cuff 3°: Lesion of the rotator cuff	1° case: Deep vein thrombosis of basilica and humeral veins on the 4th post-operative day 2° case: DVT of the popliteal vein and tibiofibular branch of leg on the 10^th ^postoperative day 3° case: Occlusion of humeral, cephalic and basilica veins on the 6^th ^post-operative day	1° case: Hereditary thrombophilia (Antiphospholipid antibody syndrome) 2° case: Hereditary thrombophilia (prothrombin promoter G20210A mutation and 4G4G promoter mutation of the plasminogen activator inhibitor) 3° case: Hereditary thrombophilia (mutation of 1691A gene for Factor V and mutation in C677T gene for methylenetetrahydrofolate reductase enzyme)

Burkhart was the first to describe a case of thromboembolism after shoulder arthroscopy, in 1990, in a 32-year-old patient treated for shoulder instability [20]. Subsequent investigations revealed the presence of a mediastinic mass, later identified as a Hodgkin's lymphoma, responsible for compression of the basilica and innominata veins. The condition was treated by surgical resection of the mass and the administration of heparin.

In 2003, Polhofer [19] described a pulmonary embolism after an arthroscopic acromionplasty procedure. The 48-year-old patient, suffering from diabetes, obesity, and affected by a tumoral lesion (enchondroma), was operated under general anesthesia, in lateral decubitus and with 3 kg traction of the arm. In view of the risk factors, prophylactic treatment with low molecular weight heparin was instituted immediately postoperatively but did not prevent the embolism. Vascular catheterization was needed to eliminate the embolism.

In 2009 Horiri [18] reported the case of a 25-year-old rugby player who developed thrombophlebitis, complicated by pulmonary embolism of the brachial vein after shoulder arthroscopy for glenohumeral instability. In this report, too, the author quoted various risk factors for VTE, such as smoking habit, a long-lasting procedure (150 minutes), lateral decubitus with 3 Kg arm traction and general anesthesia. The patient was treated with oxygen therapy and low molecular weight heparin.

Also in 2009, Bongiovanni [21] described three cases of thrombosis of the arm after arthroscopy, all with a positive history for hereditary thrombophilia. This condition had not been noted before the procedure and clinical and instrumental investigations had not indicated risk factors for DVT. The first case was a 30-year-old athletic male with shoulder instability due to a lesion of the glenoid rim and supraspinal insertion. On the 4^th ^postoperative day the patient complained of pain and swelling of the arm, shown by echo-doppler to be thrombosis of the basilica and humeral veins. Tests for hereditary thrombophilia were made and high levels of Ig anticardiolipin were found, suggesting an anti-phospholipids antibodies syndrome. The second case was a 54-year-old woman operated for a rotator cuff tear. On the 10^th ^postoperative day she developed a DVT of the popliteal vein and tibio-fibular branch. Genetic studies identified a heterozygous mutation of the prothrombin and activated plasminogen inhibitor genes. The last case was a 66-year-old man operated for a massive lesion of the rotator cuff. On the 6^th ^day he developed occlusion of the humeral, cephalic and basilica veins. Tests for thrombophilia identified a mutation of the factor V and methylenetetrahydrofolate reductase genes. In all three cases follow-up was continued for 6-12 months to check resolution of the thrombosis.

In 2009, Randelli et al.[17] published the results of an Italian multicentric study assessing the number of DVT after shoulder arthroscopy occurring in the years 2005 and 2006. The research units numbered 59 orthopedic surgeons, who recorded 6 DVT over a total of 9285 shoulder arthroscopy procedures. Pharmacological treatment was successful in all cases.

In all the above cases it is important to note that there were evident preoperative risk factors for DVT and failure to take adequate precautions could justify the onset of the subsequent complication. Instead, in our two cases there were no particular conditions that could have explained the onset of a VTE. In fact, in the first case in-depth study of the clotting factors identified a heterozygous variation of one clotting factor that is considered normal in absence of other concomitant coagulation defects. In the second, there was no acquired or congenital condition justifying a coagulation defect, apart from the arthroscopic surgical procedure, and the subsequent immobilization of the joint in a sling. Thus, the onset of the VTE, that occurred later than in the previously described cases, supports the hypothesis that the problem was caused not only by the procedure but also by the subsequent immobilization.

This underlines the importance of weighing up the advantages of prophylactic anti-thromboembolic treatment after shoulder arthroscopy. The administration of low molecular weight heparin is essential to prevent VTE in patients with several risk factors [1]. Studies in the literature advise a search for the presence of anomalous hemostasis in all patients scheduled for a shoulder arthroscopy procedure, as well as investigations of smoking habit, obesity, a neoplasm, hormone therapy, etc [18]. If several of these risk factors are present it is wise to administer routine pharmacological prophylaxis for DVT but in their absence the issue poses an interesting dilemma.

In this article we have presented two clinical cases lacking any pre-existing conditions predisposing to DVT, in whom the disease must likely be attributed to the management of the arthroscopic procedure. Although this is less invasive and safer than open surgery, it still causes hemostatic and hemodynamic alterations [25,26]. In general, the technique involves lateral decubitus with the affected limb in traction, while the postoperative protocol stipulates immobilization of the joint in a sling. Therefore, all these reported cases had shown preoperative risk factors, as Creighton et al. [22] suggested.

These conditions could be responsible for a DVT, as they have already been reported as risk factors after major surgery or severe trauma of the shoulder [27,28,24]. Other aspects that warrant further study are the influence of the type of disease to be treated, the traction weight, duration of the procedure, type and duration of the postoperative immobilization and the subsequent physiotherapy protocol.

Moreover, we advise the performance of more detailed preoperative blood tests of the clotting cascade. It seems likely that even minimal enzymatic defects, although not diagnostic for thrombophilia alone, could be responsible for thrombosis when associated with other risk factors, including surgery and subsequent immobilization.

Our study according to literature data shows that DVT adverse events after shoulder arthroscopy remain a very rare complication. However, in view of the growing number of patients undergoing this procedure, this figure is expected to rise. Surgeon should take in mind this possible complication that usually appears in the first 3 weeks after surgery, so to perform adequate anti-coagulant treatment. This complication doesn't have any influence on the final outcome of surgery. Further clinical studies are therefore warranted to assess the true risk of VTE. In fact, the presence of "minor" predisposing factors that are not routinely studied, as well as the postoperative immobilization period, are potential risk factors that, associated with the invasiveness of the arthroscopy procedure, could trigger a thromboembolism.

## Consent Statement

Written informed consent was obtained from the patient for publication of this case report and accompanying images. A copy of the written consent is available for review by the Editor-in-Chief of this journal.

## Competing interests

The authors declare that they have no competing interests. We haven't any economical founding for the realization of this article.

## Authors' contributions

RG and AN gave substantial contributes in the drafting the manuscript and in the revising it for the intellectual contents. LM and SM participated in the acquisition of data of case reports. BM gave substantial contributions to interpretation of literature review. AC participated in the analysis and interpretation of data, and reviewed the manuscript. All authors read and approved the final manuscript.

## Pre-publication history

The pre-publication history for this paper can be accessed here:

http://www.biomedcentral.com/1471-2474/11/65/prepub
